# Analyzing negative feedback using a synthetic gene network expressed in the *Drosophila melanogaster* embryo

**DOI:** 10.1186/s12918-016-0330-z

**Published:** 2016-08-31

**Authors:** Ashley A. Jermusyk, Nicholas P. Murphy, Gregory T. Reeves

**Affiliations:** 1 Department of Chemical and Biomolecular Engineering, North Carolina State University, Raleigh, NC 27606 USA; 2 Department of Chemical Engineering, University of Virginia, 102 Engineers’ Way, Charlottesville, USA

**Keywords:** Negative feedback, Canalization, Shuttling, Synthetic gene networks

## Abstract

**Background:**

A complex network of gene interactions controls gene regulation throughout development and the life of the organisms. Insights can be made into these processes by studying the functional interactions (or “motifs”) which make up these networks.

**Results:**

We sought to understand the functionality of one of these network motifs, negative feedback, in a multi-cellular system. This was accomplished using a synthetic network expressed in the *Drosophila melanogaster* embryo using the yeast proteins Gal4 (a transcriptional activator) and Gal80 (an inhibitor of Gal4 activity). This network is able to produce an attenuation or shuttling phenotype depending on the Gal80/Gal4 ratio. This shuttling behavior was validated by expressing Gal3, which inhibits Gal80, to produce a localized increase in free Gal4 and therefore signaling. Mathematical modeling was used to demonstrate the capacity for negative feedback to produce these varying outputs.

**Conclusions:**

The capacity of a network motif to exhibit different phenotypes due to minor changes to the network in multi-cellular systems was shown. This work demonstrates the importance of studying network motifs in multi-cellular systems.

**Electronic supplementary material:**

The online version of this article (doi:10.1186/s12918-016-0330-z) contains supplementary material, which is available to authorized users.

## Background

Regulation of gene expression through genetic interactions, interconnected into complex networks, is crucial to the fitness of all organisms. These genetic regulatory networks are composed of several over-represented sets of interactions, called “motifs”, which are individually amenable to study [1, 2]. Many such studies are currently being conducted using synthetic gene network motifs in single-cell systems [3–9]. Such systems are highly advantageous from a practical point of view and often shed light on the dynamic behavior of network motifs. However, this research is unable to address the question of how these networks translate into inherently multi-cellular systems such as tissue patterning, stem-cell differentiation, cancer, and wound healing systems, each of which has a spatial component. This study seeks to address how a negative feedback motif behaves in space in the developing *Drosophila melanogaster* embryo.

Negative feedback loops in biology can result in a rich diversity of phenomenological behavior (reviewed in [10]). Under some conditions, negative feedback can destabilize the output of a system and create oscillations [4, 11, 12]. Under other conditions, it may instead serve to stabilize the system against perturbations in the input signal. Negative feedback acts in this manner to control tumor suppression genes in mice as well as pluripotency and self-renewal in embryonic stem cells [13, 14]. In a spatially-distributed system, the negative feedback that occurs when a morphogen activates its own inhibitor (the “self-enhanced ligand degradation” paradigm) may add robustness to downstream gene expression patterns [15–18]. Negative feedback can also be used to limit the range or length scale of a signal. This is occurs in the JAK/STAT pathway in vertebrates [19].

Here we create a spatially distributed synthetic gene network in the early *Drosophila* embryo. We use the *bcd* 3’ UTR to express the yeast activator Gal4 in an anterior-posterior gradient [20–26]. To create a negative feedback motif, we engineered a *gal80* construct to contain three or five UAS sites (Upstream Activating Sequences), which are activated by Gal4 [27–29]. Gal80 binds to Gal4, preventing transcription of *UAS*-linked genes [30, 31].

We found that, depending on the amount of *gal4* and *gal80* present in the embryo, this negative feedback system can exhibit either an attenuation or a shuttling phenotype, in which Gal4/Gal80 binding and diffusion can extend the spatial range of Gal4 signaling [32–34]. Both mathematical modeling, as well as expression of the Gal80-binder Gal3, validate our findings [35]. This work demonstrates how in spatial systems, gene networks can produce very different outputs depending on the relative spatial domains of inputs.

## Results

### Gal4-driven lacZ expression has a graded border

**Fig. 1:**
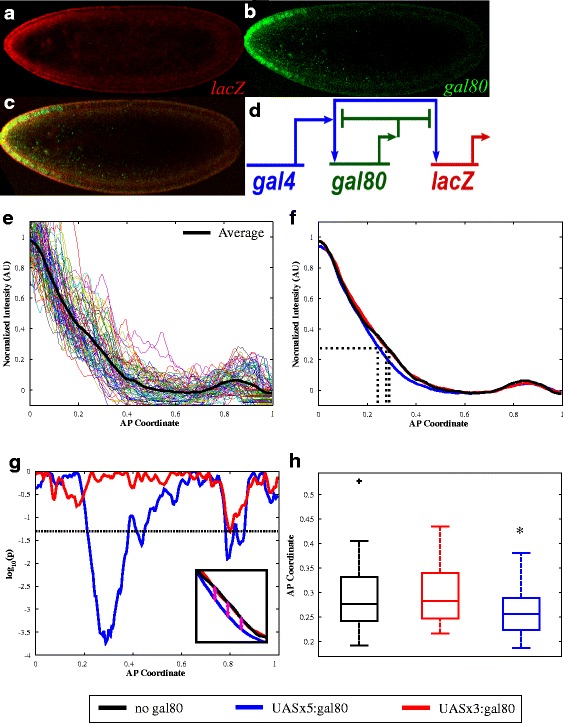
Effect of Gal80 on *lacZ* expression in attenuation situation. **a** *lacZ* mRNA expression at the mid-saggittal plane in an embryo expressing *UAS*x5:*gal80*, from mothers with four copies of Gal4GCN4. **b** *gal80* mRNA expression in the same embryo as (**a**). **c** Merged image of expression in (**a**) and (**b**). **d** Network diagram, Gal4 activated *gal80* and *lacZ* expression. Gal80 binds to Gal4, repressing *gal80* and *lacZ* activation. **e** Quantification of *lacZ* mRNA expression in embryos without *gal80* along anterior-posterior axis (given as fraction of embryo length), each colored curve represents the dorsal or ventral side of a single embryo. The average for all embryos is in black. **f** Average curves for *lacZ* expression in embryos without *gal80* (*n* = 35), with *UAS*x3:*gal80* (*n* = 36), and *UAS*x5:*gal80* (*n* = 51). Individual curves for each embryo are shown in Additional file 1: Figure S1. **g** Difference, calculated as the log_10_(p) – where p is the probability as calculated by a two-sample t-test, between the normalized intensity of *lacZ* without *gal80* versus with *UAS*x5:*gal80* or *UAS*x3*:gal80* at a given position along the anterior-posterior axis, dashed line denotes *p* = 0.05. Inset shows subset of plot in (**f**) with arrows drawn to demonstrate how the t-test is conducted at each position along AP coordinate between the applicable normalized intensity of *lacZ* with *gal80* and the control curve without *gal80*. **h** Box plots of AP coordinate where normalized intensity is 0.27 (see dashed lines in [**f**], maximum difference between no *gal80* control and *UAS*x5:*gal80* [**g**]). Asterisk denote statistical significance (*p* < 0.05)

A negative feedback network was created consisting of *gal4*, *gal80*, and *lacZ* (see Fig. 1d). We used a previously-published Gal4 construct (Gal4-GCN4:Bcd 3’UTR [23] that mimics the Bicoid anterior-posterior concentration gradient. Additionally, Gal80 should interact the same with the Gal4-GCN4 construct as with full-length Gal4 [30, 31, 36]. For baseline measurements, we first imaged embryos containing only Gal4 (four copies of this construct) and *UAS-lacZ* (no *UAS-gal80*).

In these embryos, the synthetic gradient in Gal4 activates the expression of the *UAS*-*lacZ* construct in a spatially-dependent fashion. Using fluorescent *in situ* hybridization, together with image analysis protocols (see Methods) we were able to quantify the expression domain of *lacZ* (Fig. 1e and Additional file 1: Figure S1).

We found that the expression boundary of *lacZ*, resulting from the Gal4 gradient, is not sharp, in contrast to previous work using this Gal4 construct [37]. This difference may be due to the differences between in situ hybridization procedures using alkaline-phosphatase staining versus fluorescent detection.

### Gal80 expression attenuates lacZ expression

Next, to measure the effect of the negative feedback loop, we analyzed embryos containing all three constructs: *gal4* (four copies of the *gal4-bcd 3’UTR)*, *UAS-lacZ*, as well as *UAS-gal80* (one copy). We tested two different promoter strengths for *gal80*: three or five UAS sites were used. The expression profile for *lacZ* with and without *gal80* was analyzed to determine the effect of Gal80 mediated negative feedback on *lacZ* production due to Gal4. We found that the expression pattern of *lacZ* is qualitatively unchanged (Fig. 1f). Furthermore, *gal80* expression is similar to that of *lacZ*.

To determine the extent to which *gal80* affects the *lacZ* profile, we first compared the normalized intensity of *lacZ* at each point along the anterior-posterior (AP) axis when there is no *gal80* present (control) to when there is *gal80* present (in either the three or five UAS site scenario) (Fig. 1g). There was no statistically significant difference between the normalized intensity of *lacZ* without *gal80* and with *gal80* linked to three UAS sites along the entire AP axis. However, there is a difference (*p*-value < 0.05) between the profiles for *lacZ* without *gal80* and with *gal80* linked to five UAS sites from 21 to 38 % embryo length and from 41 to 44 % embryo length; with the maximum difference at 29 % embryo length (Fig. 1g, see Additional file 2).

To compare the curves using a single summary statistic, we evaluated the AP position, *x*_*L*_, at which the *lacZ* profile fell to 27 % maximal intensity. This level was chosen because it corresponds to the normalized intensity of the *lacZ* profile with no *gal80* at 29 % embryo length. As we found previously, with only 3 UAS sites driving gal80 expression, no statistically significant effect on the *lacZ* profile was observed, as compared to the system without *gal80* (Fig. 1h).

From this analysis we are able to characterize the nature of the shift in *lacZ* when *UAS*x5:*gal80* is present in the system. In this case, attenuation is observed and *lacZ* expression was shifted toward the anterior pole (x = 0.262 ± 0.045 with UASx5:*gal80* vs. x = 0.291 ± 0.071 with no gal80, *p* = 0.038) (Fig. 1h, see Additional file 2). At the same time a decrease in the standard deviation was observed (F-test for variance, *p* = 0.002). These two observations are indicative of negative feedback and demonstrate the ability of a simple negative feedback loop to reproducibly give rise to gene expression in a given spatial domain by buffering against minor biological and environmental fluctuations.

### Increasing abundance of Gal80 creates a shuttling system

In order to increase the strength of negative feedback through Gal80 we altered the copy number of *gal4* and *gal80* transgenes in this system. We considered the possibility that only weak negative feedback was seen due to a limited amount of Gal80 protein. Therefore, we tested whether increasing the amount of Gal80 relative to Gal4 would result in a greater effect of Gal80 and enhanced control due to negative feedback on the system. We analyzed the *lacZ* profiles in embryos carrying two copies of *gal4-bcd 3’UTR* (half the number of copies of *gal4* as used previously) and either one or two copies of *UAS-gal80* (previously only one copy of *UAS-gal80* was used). We assume that two copies of the *gal80* transgene results in double the potential Gal80 protein synthesis rate. However, we do not assume that two copies of the *gal4* transgene results in one-half the amount of Gal4 protein loaded into the embryo, due to the *gal4* transgene existing at two separate genomic loci.

**Fig. 2:**
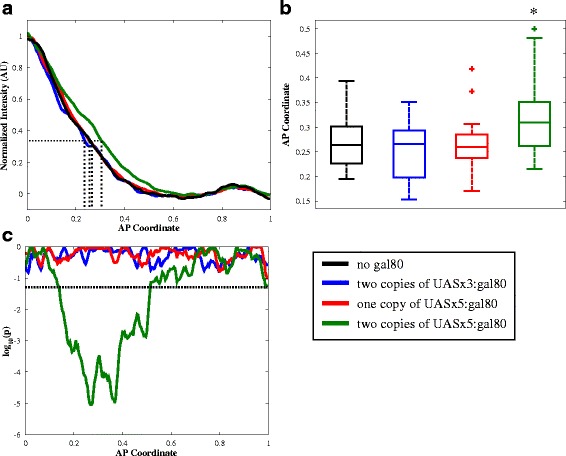
Gal80 is able to create a shuttling system. **a** Average curves of *lacZ* mRNA expression at the mid-saggittal plane in embryos with no *gal80* (*n* = 27), two copies of *UAS*x5:*gal80* (*n* = 36), two copies of *UAS*x3:*gal80* (*n* = 13), or one copy of *UAS*x5:*gal80* (*n* = 22), from mothers with two copies of Gal4GCN4. Individual curves for each embryo are shown with the average in Additional file 1: Figure S2. **b** Box plots of AP coordinate where normalized intensity is 0.31 (see dashed lines in [**a**], maximum difference between no *gal80* control and two copies of *UAS*x5:*gal80* [**c**]). Asterisk denotes statistical significance (*p* < 0.005). **c** Difference between normalized intensity of *lacZ* without *gal80* versus with varying amounts of *gal80*, dashed line denotes *p* = 0.05

As before, the *UAS-gal80* construct contained either three or five UAS sites. To quantify the differences between these curves, we determined the point along the AP axis at which each curve passes 31 % maximal intensity (Fig. 2a). This corresponds to the normalized intensity of the *lacZ* profile in embryos without Gal80 at 26 % embryo length, or the position along the AP axis with the maximum difference between embryos without Gal80 and with two copies of *UAS*x5:*gal80*. At a moderate Gal80 to Gal4 ratio (two copies of *gal4* and one of *gal80*), there was no discernible change in the lacZ profile as compared to the no-Gal80 control (Fig. 2). However, at the highest Gal80 to Gal4 ratio, the *lacZ* profile shifted away from the anterior pole (x = 0.321 ± 0.073 vs. x = 0.265 ± 0.046 with no gal80, *p* = 5 × 10^−4^), contrary to expectation (see Fig. 2b, see Additional file 2).

**Fig. 3:**
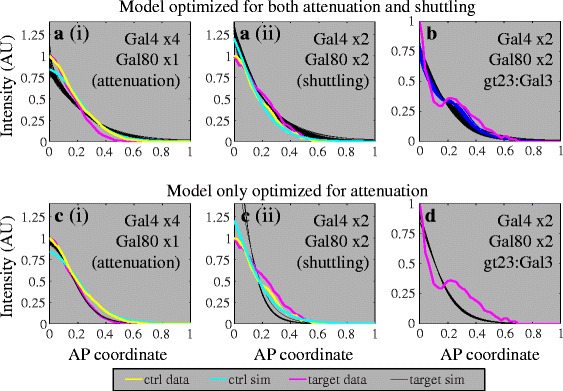
A mechanistic model of Gal4/Gal80/Gal3 interactions supports our hypothesis. **a** The model, when simultaneously fit to both the attenuation (i) and shuttling (ii) data, is able to adequately satisfy both scenarios. The same parameter sets were used in both (i) and (ii), with the only difference being that the levels of Gal4 and Gal80 are altered. The experimentally determined expression of *lacZ* due to Gal4 without Gal80 is shown in yellow (ctrl data), the simulation fit to these data is shown in cyan (ctrl sim). The expression of *lacZ* after the addition of Gal80 found through experiments is shown in magenta (target data), the simulation fits to these data are shown in black (target sim). **b** When Gal3 is added to the system, the model exhibits a similar phenotype as experimentally observed when the model is optimized for both attenuation and shuttling. The parameter sets here are the same as shown in (**a**). **c** When the model is fit only to the attenuation phenotype, the attenuation fit is better (i), but shuttling does not occur (ii)**. d** With parameter sets that resulted from an attenuation-only optimization, as seen in (**c**), the presence of Gal3 does not result in a local increase in *lacZ* expression. The parameter sets here are the same as shown in (**c**)

**Fig. 4:**
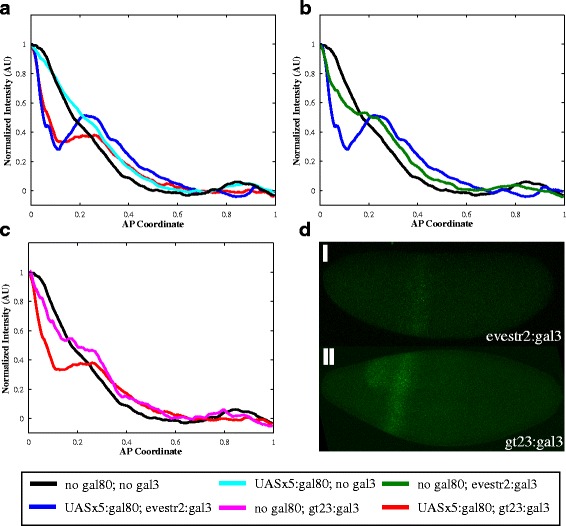
Localized Gal3 creates a peak in *lacZ*. **a** Average curves of *lacZ* expression in embryos without *gal80* (*n* = 27) and with two copies of *UAS*x5:*gal80* with no *gal3* (*n* = 36), *evestr2*:*gal3* (*n* = 19), and *gt23*:*gal3* (*n* = 12). **b** Average curves of *lacZ* expression in embryos without *gal80* and without *gal3*, without *gal80* and with *evestr2:gal3* (*n* = 9), and with *gal80* and *evestr2:gal3.* **c** Average curves of *lacZ* expression in embryos without *gal80* and no *gal3*, without *gal80* and with *gt23:gal3* (*n* = 6), and with *gal80* and *gt23:gal3*. **d** Expression of *gal3* in embryos expressing (i) *evestr2*:*gal3* and (ii) *gt23*:*gal3*

One phenomenon that could be responsible for expansion of the lacZ profile is facilitated diffusion or “shuttling” [32–34]). This “shuttling” would occur if Gal80 binding to Gal4 increases the effective diffusion of Gal4. This can occur if the Gal4/Gal80 complex exists preferentially unbound to the DNA as compared to Gal4, which would result if Gal80 destabilizes Gal4-DNA binding. The existence of this shuttling phenomenon was validated in a number of ways: using a model to demonstrate it is biophysically possible to switch between attenuation and shuttling in our system (Fig. 3a), adding a molecule to break-up this Gal4/Gal80 complex and create an effective sink for Gal4 (Figs. 3b and 4a), and showing that shuttling is required to observe this increase in signaling when using this molecule to break-up the Gal4/Gal80 complex (Fig. 3d).

### A model of Gal4/Gal80 interactions predicts both attenuation and shuttling regimes

One question that arises is whether it would be biophysically possible to have a system in which Gal80 attenuates the signaling range of Gal4 at low Gal80:Gal4 ratios, but extends the signaling range of Gal4 at high Gal80:Gal4 ratios. To answer this question, we built a mechanistic model of Gal4/Gal80 interactions. To simplify this model, we consider a lumped model for cytoplasmic, nuclear, and DNA-bound Gal4 and Gal4/Gal80 complex, and similarly lump cytoplasmic and nuclear Gal80. The effect of Gal80 binding to Gal4 has on the interaction between Gal4 and the DNA is accounted for in the diffusion term for Gal4. These equations are shown below.

$$ \begin{array}{c}\hfill 0={\lambda}_g^2{g}_{xx}-g-\mu \left(gr-\nu c\right)\hfill \\ {}\hfill 0={\lambda}_r^2{r}_{xx}-r-\beta \mu \left(gr-\nu c\right)+{q}_r{f}_r(g)\hfill \\ {}\hfill 0={\lambda}_c^2{c}_{xx}-{\rho}_cc+\mu \left(gr-\nu c\right)\hfill \end{array} $$

In these equations, *g* represents the concentration of Gal4, *r* the concentration of the repressor Gal80, and *c* the Gal4/Gal80 complex. At steady state, each component diffuses, is degraded, and participates in a reversible binding reaction with forward rate *μ* and affinity *v*. Gal80 is produced by nuclei in which Gal4 signaling, represented by the function *f*_*r*_(*g*) (see Methods), is sufficiently high. We assume no-flux boundary conditions for all species at both *x* = 0 (anterior pole) and *x* = 1 (posterior pole), except for a constant flux production of *g* at *x* = 0 (see Additional file 1).

The constant flux production of Gal4 at the anterior pole is denoted by the parameter *q*_*g*_, which equals 1 for four copies of Gal4, and *q*_2*x*_ for two copies. As mentioned above, because the copies of *gal4* are at two different sites within the genome, we could not be sure that two copies of *gal4* resulted in one half the production of Gal4 protein as compared to four copies of *gal4*. Therefore, we investigated the behavior of the model for *q*_2*x*_ between 0.35 and 0.65. We found that the model was better able to fit our data for lower values of *q*_2*x*_, so all results displayed are for *q*_2*x*_ = 0.35.

To fit our model to the experimental data, we first examined the case with no *gal80* (*q*_*r*_ = 0). We fit this version of the model to our data with no *gal80* (ctrl data; yellow curves in Fig. 3) and found adequate fits in both the attenuation case with Gal4x4 (*q*_*g*_ = 1; cyan curve in Fig. 3a(i)) and the shuttling case with Gal4x2 (*q*_*g*_ = *q*_2*x*_; cyan curve in Fig. 3a(ii)).

Next, we fit our model to the data from embryos carrying *gal80* (target data; magenta curves in Fig. 3). We performed this fit simultaneously (see Additional file 1) under two conditions: with *q*_*r*_ = 1 and *q*_*g*_ = 1 (for four copies of *gal4* and one copy of *gal80*; Fig. 3a(i)) and *q*_*r*_ = 2, *q*_*g*_ = *q*_2*x*_ (for two copies of *gal80* and two copies of *gal4*; Fig. 3a(ii)). We found the model was able to adequately reproduce both an attenuated *lacZ* profile in the Gal4x4/Gal80x1 scenario (target sim; family of black curves in Fig. 3a(i)), as well as an expanded *lacZ* profile in the Gal4x2/Gal80x2 scenario (target sim; family of black curves in Fig. 3a(ii)). This supports the plausibility of the hypothesis that the system performs attenuation for one dosage ratio and shuttling for another.

### Expression of Gal3 in a stripe results in a peak of lacZ expression

One prediction of the shuttling hypothesis is that forcing the local degradation or capture of the inhibitor (Gal80) should result in a similarly localized peak in signaling activity. Therefore, we introduced the yeast protein Gal3 into the Gal4/Gal80 system. Gal3 binds to Gal80 and prevents the formation of the Gal4/Gal80 complex, thereby allowing Gal4 to activate UAS-linked genes [35, 38, 39].

Two different enhancer regions were used to create stripes of Gal3 (Fig. 4), namely *gt23* [40] and *evestr2* [41]. As predicted by the shuttling hypothesis, the expression of Gal3 in these domains causes a local increase in *lacZ* production (Fig. 4). This perturbation also causes a rapid decrease in lacZ expression anterior to the *gal3* expression domain. This is likely due to the increase in concentration of free Gal4 within the *gal3* domain at the expense of Gal4 outside of this domain.

To control for the possibility that Gal3 expression is causing UAS-*lacZ* transcription without Gal80, we examined embryos that carried two copies of Gal4 and Gal3, but lacked Gal80. Surprisingly, a small increase in *lacZ* expression near the site of Gal3 expression was also observed when no Gal80 was present in the system (Fig. 4c). While the yeast literature does not suggest that Gal3 interacts with Gal4, this increase in *lacZ* expression (Fig. 4b, c) may be due to some previously unknown interaction with Gal4. However, Gal3 has a more significant localization effect when Gal80 is in the system, supporting our hypothesis that Gal80 shuttles Gal4 (see Additional file 1: Figure S3).

To confirm that increased *lacZ* expression in the Gal3 domains is explained only by the shuttling phenomenon, we extended our model to include the presence of Gal3 (see Additional file 1). When the model is optimized to simultaneously fit both the attenuation and shuttling regimes (described in the previous section and depicted in Fig. 3a), local Gal3 expression can result in a corresponding local increase in *lacZ* expression (families of black and blue curves in Fig. 3b). Note that, in addition to an increase in *lacZ* output in the *gal3* domain, the better-fit curves (blue) also fit a secondary detail of our experimental data: a more rapid decrease in *lacZ* anterior to the *gal3* domain. These results show that a model in which Gal80 shuttles Gal4 is consistent with our Gal3-induced *lacZ* data.

Next, we asked whether the shuttling phenomenon is required by our model to fit the Gal3-induced *lacZ* data. To answer this question, we fit the Gal4/Gal80 model to only the Gal4x4/Gal80x1 scenario (attenuation; Fig. 3c(i)). This corresponds to setting the parameter *x* = 0 (explained in Additional file 1). Note that, with these parameter choices that are optimized only for attenuation, and not for shuttling, our shuttling target simulation curves (family of black cuves in Fig. 3c(ii)) do not match the target data (magenta curve in Fig. 3c(ii)). In this case, the presence of Gal3 does not alter the *lacZ* expression profile (Fig. 3d). This shows that the shuttling phenomenon is not only sufficient (as described in the previous paragraph), but also necessary in order to match the phenotype observed in our experiments. Taken together, our Gal3 experimental results unambiguously support the presence of shuttling within our system.

## Discussion

A synthetic negative feedback network consisting of *gal4*, *gal80*, and *lacZ* was expressed in the *Drosophila* embryo. This experimental system was able to produce weak negative feedback, marked by attenuation, the shifting of expression of *lacZ* toward the source of activation (anterior pole) and a lowered variability. When the copy numbers of *gal4* and *gal80* were altered, a shuttling system was created at a high Gal80 to Gal4 ratio. In this shuttling system, the *lacZ* profile expands toward the posterior pole, away from the source of activation. Shuttling has been found in other systems, and in some cases produces robust gradients from an initial broad morphogen signal [32–34]. Shuttling requires a diffusible morphogen and a shuttling molecule that forms a complex with, and thereby extends the spatial range of, the morphogen. In this case the shuttling molecule, Gal80, is activated by the morphogen, Gal4. A third molecule can be used which breaks up the shuttling molecule/morphogen complex, releasing the active morphogen. We were able to introduce Gal3, which results in an increase in *lacZ* expression at the source of Gal3. This is caused by the freeing of Gal4 from the Gal4/Gal80 complex. This provides further evidence for our shuttling system.

Previous studies have found evidence for morphogen gradients which enhance their own degradation, this form of negative feedback is known as self-enhanced ligand degradation. In this system morphogens degrade at a fast rate where their levels are highest and at a slower rate farther from its source, increasing the signaling range for the morphogen. This has been found in Wingless and Hedgehog patterning in the *Drosophila* wing, BMP signaling and DV axis specification in the zebrafish and *Xenopus* embryos, Wnt and EGFR signaling systems in mammalian cells and *Drosophila* embryos, and retinoic acid signaling in zebrafish [15, 18, 42–46].

Parallels from these systems can be drawn to our synthetic system. From our synthetic system we can understand in isolation the mechanisms at work in these systems better. Namely, how the simple negative feedback system is able to achieve both an attenuation and a shuttling system depending on the relative concentrations of components in the pathway. Further quantitative studies of this system (such as measurements of Gal4/Gal80 protein ratios, Gal4 and Gal80 diffusivities, and protein/mRNA spatiotemporal dynamics) would help uncover the precise mechanism for switching between an attenuation regime and a shuttling regime.

## Conclusions

While shutting has only recently been proposed to explain the ability of certain morphogen gradients to be defined and achieve robust patterns, comparisons to other systems suggest that shuttling may exist in other negative feedback systems [33]. Most importantly this work demonstrated a negative feedback system that is able to produce two very different outputs depending on the spatial domains of expression and relative amounts of these genes. This shows the complexity of gene networks in tissue patterning and other multi-cellular systems. While much previous work has been carried out to understand synthetic gene networks in single-celled systems, much care must be taken to extrapolate these findings into multi-cellular systems.

## Methods

### Plasmids

All plasmids were constructed from the pUAST parent plasmid (gift from J. Mahaffey). The *UAS*x5:*gal80* was created by inserting gal80 (PCR amplified from genomic DNA of flies containing gal80) into the pUAST digested with *Not*I and *Xba*I. The *UAS*x3 construct was obtained by inserting the *UAS* sequence (TGCGGAGTACTGTCCTCCGAG) into pBlueScript II SK (+) (from Addgene) flanked by *Sal*I and *Xho*I restriction sites. Subseqent multimerization was performed using restriction digest with *Sal*I/*Not*I and *Xho*I/*Not*I and subsequent ligation. The final *UAS*x3:*gal80* plasmid was created by insertion of *UAS*x3 (PCR amplified from *UAS*x3 in pBlueScript), *hsp70* (PCR amplified from pUAST), and *gal80* into pUAST parent plasmid. The *gal3* constructs were made by inserting the *eve* minimal promoter (PCR amplifed from genomic DNA), *gal3* (PCR amplified from yeast genomic DNA), and either *evestr2* (PCR amplified from genomic *Drosophila* DNA using primers AGATACATaagcttGCCATCAGCGAGATTATTAGTCAA and AGACTCAGctgcagAGGGCTAAGTCGGCGCAAA) or *gt23* (PCR amplified using primers AGATCATaagcttGGGAATTCGGCGACTTGGATCGTGAG and ATGACACActgcagAAAACTGCAGCTGCCCTGCCCTGCTCTG from genomic *Drosophila* DNA) enhancer regions into the *UAS*x3:*gal80* plasmid.

### Fly stocks

The w-;Gal4-GCN4:Bcd 3’UTR;Gal4-GCN4:Bcd 3’UTR flies were a gift from Dr. Dostatni [23]. The *UASp*:*lacZ* flies used in this study were obtained from Bloomington Stock Center (BS 3955). The *UAS*x3:*gal80* and *UAS*x5:*gal80* fly lines were created by injection and incorporation of plasmid constructs into the 68A4 landing site (injections performed by Model System Injections into yw;attP2 flies). The *gal3* constructs were incorporated into the 65B2 landing site by fly injection (injections performed by Model System Injections into yw;VK33 flies).

### Embryo staining and image collection and analysis

Embryos were fixed 2–4 h after egg laying per standard protocols. Fluorescent *in situ* hybridization was conducted per published protocols [47] (Proteinase K treatment was omitted) using RNA probes for *lacZ* (biotin conjugated), *gal80* (flourescein conjugated), and *gal3* (digoxigenin conjugated). Primary antibodies to biotin (goat anti-biotin, 1:5000; gift from Immunoreagents), flourescein (rabbit anti-fluorescein, 1:500; ThermoFisher Scientific), and digoxigenin (mouse anti-digoxigenin, 1:500; Roche). Secondary antibodies used were Alexa Flour 488 donkey anti-rabbit (ThermoFisher Scientific), Alexa Flour 546 donkey anti-goat (ThermoFisher Scientific), and Alexa Flour 488 donkey anti-mouse (ThermoFisher Scientific).

Images were taken at the mid-saggittal plane (determined optically) using a Zeiss Confocal microscope.

The images were analyzed using a modified version of the method described in [48]. The code was altered to fit the mid-saggittal section of the embryo to an ellipse using elipse_fit.m (written by Tal Henel, available from Matlab Central) using the two foci of the ellipse, the embryo was broken up into two half circles (at the anterior and posterior poles) and a rectangle bridging the two circles. The flourescence intensity around the periphery of the embryo was determined in each section (code available). See Additional file 1 for more details.

Once the images were analyzed, the raw curves were first averaged together to produce a “canonical” profile (see [48]). The canonical profile was then used as a template to align the single-embryo curves, minimizing the difference among the curves. The aligned curves were then averaged to create the profiles to which the model was fit.

## Additional files

Additional file 1: Supplemental figures and model equations. (DOCX 12250 kb) Additional file 2:
*lacZ* expression data. Experimental data of normalized *lacZ* expression with different levels of Gal4 and Gal80. (XLS 102 kb)
