# Supplementary material for: Analyzing negative feedback using a synthetic gene network expressed in the Drosophila melanogaster embryo
Source: BMC Syst Biol. 2016 Aug 31;10(1):85. doi: 10.1186/s12918-016-0330-z (PMC5006508; doi:10.1186/s12918-016-0330-z)
Supplement: Additional file 1: — Supplemental figures and model equations. (DOCX 12250 kb) [file 12918_2016_330_MOESM1_ESM.docx]

**Additional file 1: Supplemental Figures and Model Formation**

**
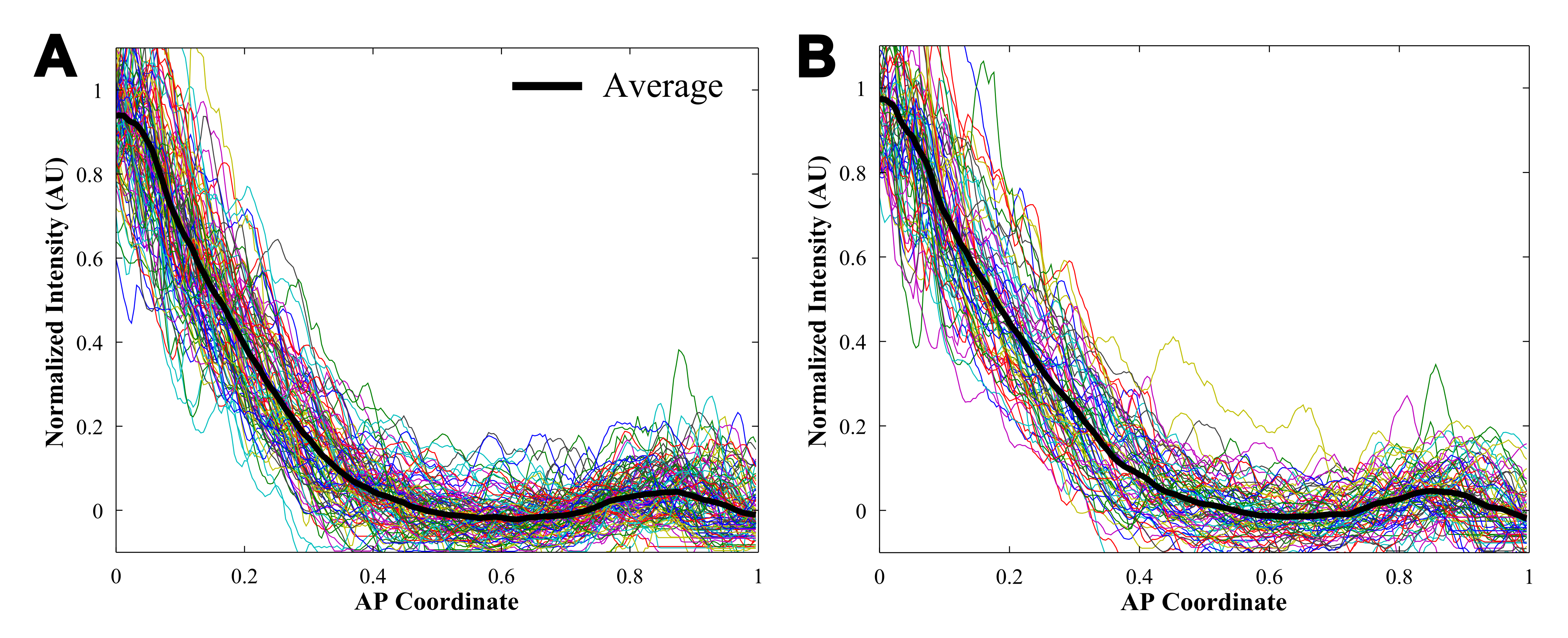
Figure S1:** ***lacZ* expression in embryos with four copies of gal4 and one copy of gal80.**

Each colored curve is the ventral or dorsal side of an individual embryo: **(A)** *UAS*x5:*gal80* and **(B)** *UAS*x3:*gal80*.

**
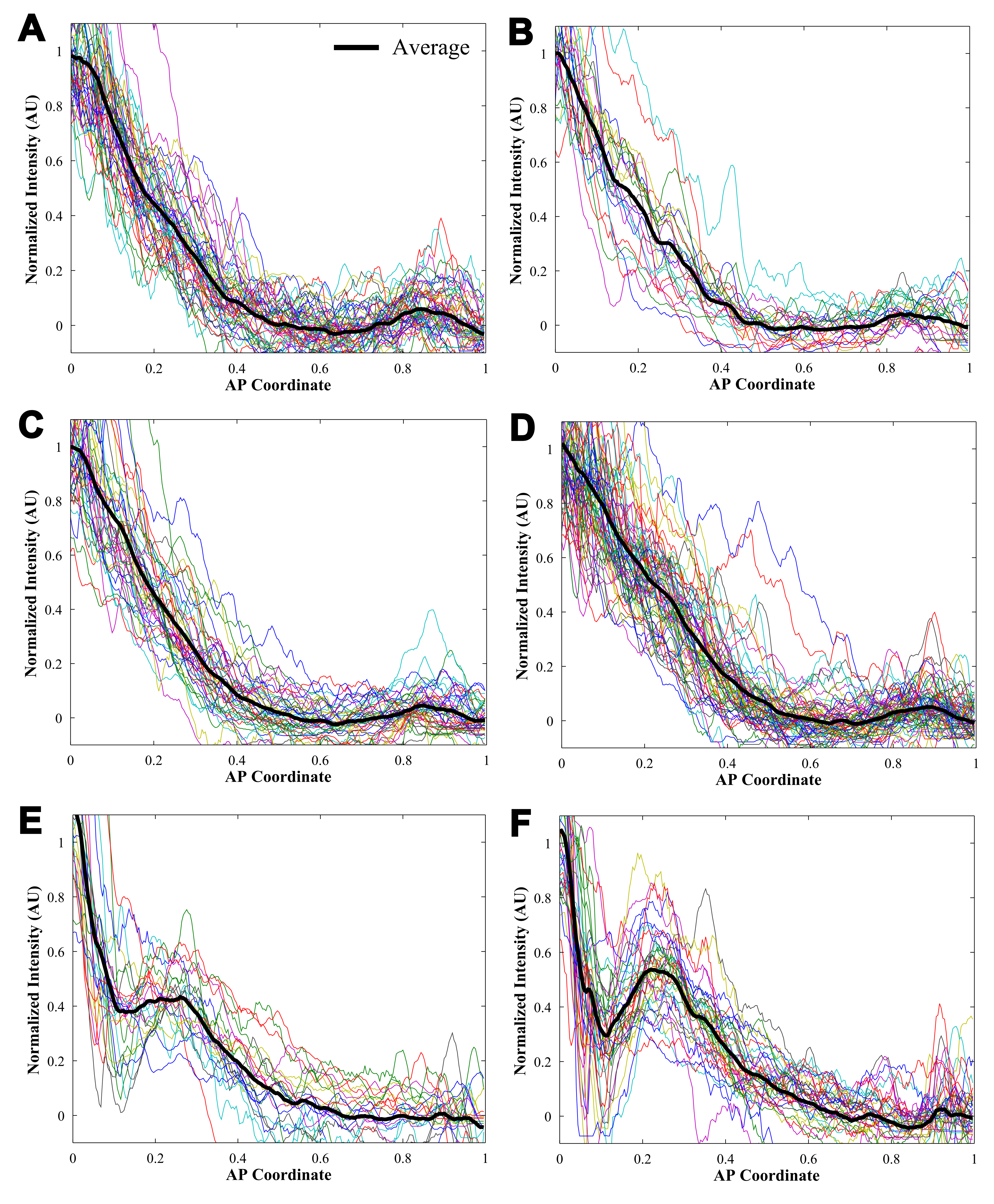
**

**Figures S2: *lacZ* expression in embryos with two copies of gal4.**

Each colored curve is the ventral or dorsal side of an individual embryo: **(A)** no gal80, **(B)** two copies of *UAS*x3:*gal80*, **(C)** one copy of *UAS*x5:*gal80*, **(D)** two copies of *UAS*x5:*gal80* (no *gal3*), **(E)** two copies of *UAS*x5:*gal80* and *gt23*:*gal3*, **(F)** two copies of *UAS*x5:*gal80* and *evestr2:gal3*.


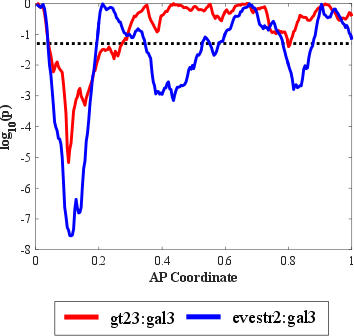


**Figures S3: Difference between *lacZ* expression in embryos expressing Gal3 with and without Gal80.**

The results of a two-sample t-test between the normalized intensity of *lacZ* with two copies of *UAS*x5:*gal80* or no *gal80* (with both containing *gal3*) at a given position along the anterior-posterior axis. The dashed line denotes p = 0.05.

### Gal4/Gal80 Model formulation

The model was formulated with the following equations corresponding to Gal4, Gal80, and Gal4/Gal80 complex, respectively:

$$\frac{\partial G}{\partial t}=D_{G}\frac{\partial^{2}G}{\partial X^{2}}-k_{G}G-k_{\mathrm{bind}}\left( GR-K_{D}C \right) (S1)$$

$$\frac{\partial R}{\partial t}=D_{R}\frac{\partial^{2}R}{\partial X^{2}}-k_{R}R-k_{\mathrm{bind}}\left( GR-K_{D}C \right)+Q_{R}F_{R}(G) (S2)$$

$$\frac{\partial C}{\partial t}=D_{C}\frac{\partial^{2}C}{\partial X^{2}}-k_{C}C+k_{\mathrm{bind}}\left( GR-K_{D}C \right) (S3)$$

For simplicity, we lump three species of Gal4 (cytoplasmic, nuclear, and DNA-bound) into one equation (Eq S1). We also lump cytoplasmic and nuclear Gal80 into Eq S2. Finally, we lump three species of Gal4/Gal80 complex cytoplasmic, nuclear, and DNA-bound) into one equation (Eq S3). These equations are subject to no flux boundary conditions at $X=0$ (anterior pole) and $X=L$ (posterior pole), except for the flux of Gal4 at the anterior pole is:

$$D_{G}\left. \frac{\partial G}{\partial X} \right|_{X=0}=-Q_{G}$$

The production of Gal80, as a function of Gal4, is as follows [1]:

$$F_{R}\left( G \right)=\frac{\left( 1+G/{K_{\mathrm{DNA}}} \right)^{n}-1}{\left( 1+G/{K_{\mathrm{DNA}}} \right)^{n}}$$

Here $n=5$ for *UAS*x5:*gal80*.

### Scaling

Assuming steady state, we next scale these equations by the following transformations:

$$g=G/\bar{G},r=R/\bar{R},c=C/\bar{C},x=X/L$$

The concentration scales are defined as follows:

$$\bar{G}=\frac{Q_{G,0}}{k_{G}L}, \bar{R}=\frac{Q_{R,0}}{k_{R}},\bar{C}=\bar{G}$$

Here $Q_{G,0}$ is the flux of Gal4 that results from translation at the anterior pole in Gal4x4 embryos, and $Q_{R,0}$ is the maximal production rate of Gal80 in embryos carrying on copy of *gal80*. This scaling results in the equations found in the main text. The constant flux boundary condition becomes

$${\lambda_{g}^{2}\left. \frac{\mathrm{dg}}{\mathrm{dx}} \right|}_{x=0}=-q_{g}$$

And the production function for Gal80 becomes:

$$f_{R}\left( g \right)=\frac{\left( 1+g/K \right)^{n}-1}{\left( 1+g/K \right)^{n}}$$

with the following parameter definitions:

$$\lambda_{g}^{2}=\frac{D_{G}}{k_{G}L^{2}},\lambda_{r}^{2}=\frac{D_{R}}{k_{R}L^{2}},\lambda_{c}^{2}=\frac{D_{C}}{k_{G}L^{2}}$$

$$\mu=\frac{k_{\mathrm{bind}}\bar{R}}{k_{G}},\nu=\frac{K_{D}}{\bar{R}},\beta=\frac{k_{G}\bar{G}}{k_{R}\bar{R}}$$

$$\rho_{c}=\frac{k_{C}}{k_{G}},q_{r}=\frac{Q_{R}}{Q_{R,0}},q_{g}=\frac{Q_{G}}{Q_{G,0}},K=\frac{K_{\mathrm{DNA}}}{\bar{G}}$$

### Gal3

When considering Gal3, the following equations (for Gal3 and Gal3/Gal80 complex, respectively) were added to the model:

$$\frac{\partial B}{\partial t}=D_{B}\frac{\partial^{2}B}{\partial X^{2}}-k_{B}B-k_{3}\left( BR-K_{3}P \right)+Q_{B}F_{B}(X)$$

$$\frac{\partial P}{\partial t}=D_{P}\frac{\partial^{2}P}{\partial X^{2}}-k_{P}P+k_{3}\left( BR-K_{3}P \right)$$

Additionally, the term $-k_{3}\left( BR-K_{3}P \right)$ also is included in the equation for Gal80. These equations are both subject to no-flux boundary conditions at $X=0$ and $X=L$. The production term for Gal3, $F_{B}(X)$, reflects the localization of Gal3 from the gt23 enhancer (see Sup Fig. S4).


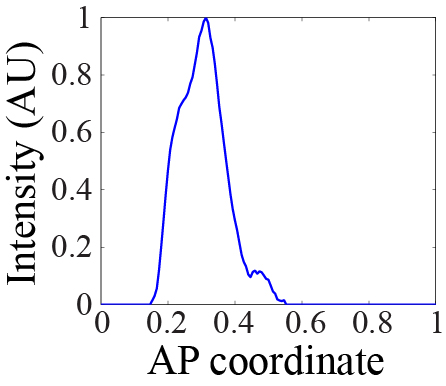


**Figure S4: Localization of *gt23*:*gal3* expression.**

To scale these equations, we made the following transformations (at steady state):

$$b=B/\bar{B}, p=P/\bar{P}$$

The concentration scales are defined as follows:

$$\bar{B}=\frac{Q_{B,0}}{k_{B}L}, \bar{P}=\bar{R}$$

Here $Q_{B,0}$ is the maximal production rate of Gal3 in embryos carrying on copy of *gt23*:*gal3*. This scaling results in the following equations:

$$0=\lambda_{b}^{2}\frac{d^{2}b}{dx^{2}}-b-\eta\gamma\left( br-\sigma p \right)+q_{b}f_{b}(x)$$

$$0=\lambda_{p}^{2}\frac{d^{2}p}{dx^{2}}-\rho_{p}p+\eta\left( br-\sigma p \right)$$

with the following parameter definitions:

$$\lambda_{b}^{2}=\frac{D_{B}}{k_{B}L^{2}},\lambda_{p}^{2}=\frac{D_{P}}{k_{R}L^{2}}$$

$$\eta=\frac{k_{3}\bar{B}}{k_{R}},\sigma=\frac{K_{3}}{\bar{B}},\gamma=\frac{k_{R}\bar{R}}{k_{B}\bar{B}}$$

$$\rho_{p}=\frac{k_{P}}{k_{R}},q_{b}=\frac{Q_{B}}{Q_{B,0}}$$

### Optimization of the model to the observed *lacZ* profiles

Our optimization methodology proceeded as follows. First, a value of $q_{2x}$ is chosen. Next, the best-fit values for $\lambda_{g}$ and $K$ are found by fitting the model to the *lacZ* data in embryos that lack *gal80*. Next, we use evolutionary optimization [2, 3] to fit the model to the *lacZ* data for embryos with *gal80* (but without *gal3*). This gives us the further parameters $\lambda_{r}$, $\lambda_{c}$, $\mu$, $\nu$, $\beta$, and $\rho_{c}$. Finally, we use the *lacZ* data from embryos with *gal80* and *gt23*:*gal3* to constrain the parameters associated with Gal3. After these fits are found, the value of $q_{2x}$ is varied (as 0.35, 0.40, 0.45, … 0.65) and we run the procedure again. Details follow.

#### Optimization of embryos with no gal80

The first set of optimizations were performed on the *lacZ* data in embryos with no *gal80*. The model without Gal80 reduces to a single equation with only three unknown parameters: $\lambda_{g}$, $K$, and $q_{2x}$. $q_{2x}$ was chosen as described above as an input to the optimization procedure. For a given choice of $q_{2x}$, $\lambda_{g}$ was varied on an equally-spaced grid with 31 points from 0.05 to 0.2, and the Gal4 equation was solved for Gal4 x4 ($q_{r}=1$) and Gal4 x2 $(q_{r}=q_{2x}$). Next, $K$ was varied on a log-space with 200 points from 0.1 to 1000. The Gal4 gradients were then used to compute the *lacZ* profiles in both cases (where the *lacZ* profile was equal to $f_{r}(g)$). The cost function was defined as follows:

$$F=\sum_{i=a}^{i=b} f_{4x,i}^{2}+f_{2x,i}^{2}$$

where $a$ is the index for $x_{i}=0.1$, and $b$ is the index for $x_{i}=0.9$ (we limit ourselves to the central 80% of the embryo to avoid curvature effects). The cost coefficient for Gal4 x4 embryos is

$$f_{4x,i}=\frac{Y_{4x,i}-\alpha_{0}L_{4x,i}}{S_{4x,i}}$$

and where $Y_{4x,i}$ is the value of the observed mean *lacZ* profile at $x_{i}$ in Gal4 x4 embryos, $L_{4x,i}$ is the value of the simulated *lacZ* profile at $x_{i}$ in Gal4 x4 embryos, and $S_{4x,i}$ is the standard deviation of the observed *lacZ* profiles at $x_{i}$ in Gal4 x4 embryos. $\alpha_{0}$ is a scaling constant that minimizes the difference between $Y_{4x}$ and $L_{4x}$ for $x$ between $x_{a}$ and $x_{b}$.

Similarly, the cost coefficient for Gal4 x2 embryos is

$$f_{2x,i}=\frac{Y_{2x,i}-{\alpha_{0}L_{2x,i}}/{\alpha_{2x}}}{S_{2x,i}}$$

where $Y_{2x,i}$ is the value of the observed mean *lacZ* profile at $x_{i}$ in Gal4 x2 embryos, $L_{2x,i}$ is the value of the simulated *lacZ* profile at $x_{i}$ in Gal4 x2 embryos, and $S_{2x,i}$ is the standard deviation of the observed *lacZ* profiles at $x_{i}$ in Gal4 x2 embryos. $\alpha_{2x}$ is a scaling constant that minimizes the difference between $Y_{2x}$ and $\alpha_{0}L_{2x}$ for $x$ between $x_{a}$ and $x_{b}$.

Using this procedure, we found the best-fit $\lambda_{g}$ and $K$ for every value of $q_{2x}$ that served as input.

#### Optimization of embryos with gal80 but no gal3

The next set of optimizations were performed on the *lacZ* data in embryos with *gal80* but no *gal3*. The model with Gal80 has the additional parameters $\lambda_{r}$, $\lambda_{c}$, $\mu$, $\nu$, $\beta$, and $\rho_{c}$. ($\lambda_{g}$ and $K$ were held fixed at their best-fit values from the above optimization.)

We used an improved stochastic evolutionary optimization with penalty constraints (see [2, 3]) to find a cloud of parameter sets that best- fit the *lacZ* data from embryos with gal80 but no *gal3*. For each input value of $q_{2x}$, we found 100 evolutionary-optimized parameter sets.

The cost function was defined as follows:

$$F=\sum_{i=a}^{i=b} (1-\chi)f_{1x80,i}^{2}+\chi f_{2x80,i}^{2}$$

where $a$ and $b$ are described above. The cost coefficient for Gal4 x4/Gal80 x1 embryos is

$$f_{1x80,i}=\frac{Y_{1x80,i}-{\alpha_{0}L_{1x80,i}}/{\alpha_{1x80}}}{S_{1x80,i}}$$

and where$Y_{1x80,i}$ is the value of the observed mean *lacZ* profile at $x_{i}$ in Gal4 x4/Gal80 x1 embryos, $L_{1x80,i}$ is the value of the simulated *lacZ* profile at $x_{i}$ in Gal4 x4/Gal80 x1 embryos, and $S_{1x80,i}$ is the standard deviation of the observed *lacZ* profiles at $x_{i}$ in Gal4 x4/Gal80 x1 embryos. $\alpha_{1x80}$ is a scaling constant that minimizes the difference between $Y_{1x80}$ and $\alpha_{0}L_{1x80}$ for $x$ between $x_{a}$ and $x_{b}$.

The cost coefficient for Gal4 x2/Gal80 x2 embryos is analogous. The parameter $\chi$ is a scaling parameter that describes the weighting between the two sets of embryos. We chose $\chi=0.5$ (for optimization of both scenarios) and $\chi=0$ (for optimization of attenuation only).

Using this procedure, we found clouds of optimized parameter sets for the Gal80 parameters.

#### Optimization of embryos with gal80 and gal3

The final optimization was performed on the lacZ data in embryos with both *gal80* and *gal3*. The model with Gal80 and Gal3 has the additional parameters $\lambda_{b}$, $\lambda_{p}$, $\eta$, $\sigma$, $\gamma$, and $\rho_{p}$. To simplify the optimization, we assumed both diffusion length scales were zero, that non-specific degradation of the Gal3/Gal80 complex was equal to that of Gal80 alone (so that $\rho_{p}=1$), and that relatively tight binding occurs ($\sigma=0.01$). These assumptions left us with two parameters to vary for the optimization: the forward binding rate for Gal3 and Gal80, $\eta$, and the ratio of maximal production rates of Gal80 to Gal3, $\gamma$.

For each parameter set found by evolutionary optimization above, we used brute-force optimization to determine the best-fit $(\eta,\gamma$) pair. $\gamma$ was varied on an log-spaced grid with 31 points from 10^-1^ to 10^2^, and $\eta$ was varied on a log-space with 51 points from 10^0^ to 10^6^.

The cost function was defined as follows:

$$F=\sum_{i=a}^{i=b} f_{3,i}^{2}$$

where $a$ and $b$ are described above. The cost coefficient is

$$f_{3,i}=\frac{Y_{3,i}-{\alpha_{0}L_{3,i}}/{\alpha_{3}}}{S_{3,i}}$$

and where$Y_{3,i}$ is the value of the observed mean *lacZ* profile at $x_{i}$ in Gal4 x2/Gal80 x2/Gal3 x1 embryos, $L_{3,i}$ is the value of the simulated *lacZ* profile at $x_{i}$, and $S_{3,i}$ is the standard deviation of the observed *lacZ* profiles at $x_{i}$. $\alpha_{3}$ is a scaling constant that minimizes the difference between $Y_{3}$ and $\alpha_{0}L_{3}$ for $x$ between $x_{a}$ and $x_{b}$.

### Finding the boundary of embryos in images taken at the mid-saggital plane

The boundary of the embryo was found using the following procedures. First, the image (taken as described in Experimental Procedures) were background subtracted assuming the mode of the image corresponded to zero fluorescence levels. Next, the intensities from all color channels were summed. The resulting image was Gaussian filtered in both the x and y directions using ten pixels as a kernel. This created an image I1 with the embryo as a single, bright object to facilitate discovery of the embryo boundary.

Next, the center of the image was assumed to be the approximate center of the embryo. The first approximation of the boundary of the embryo was found by previously published methods [4, 5]. Briefly, we divided the image into 60 slices centered at the approximate center. The average intensity of each slice, as a function of the distance from the approximate center of the embryo, was found. The boundary within that slice was taken to be the location where the intensity dropped to 10% of the maximum value within that slice.

This approximate boundary defined a 60-point polygon, and the ellipse with the same first and second moments as the solid fill of that polygon was computed. The two foci of that ellipse then served as points about which the image I1 could be divided into four regions: two rectangular regions that each have one side as the line segment between the foci, and the other two semi-circle regions as the remaining parts of the image.


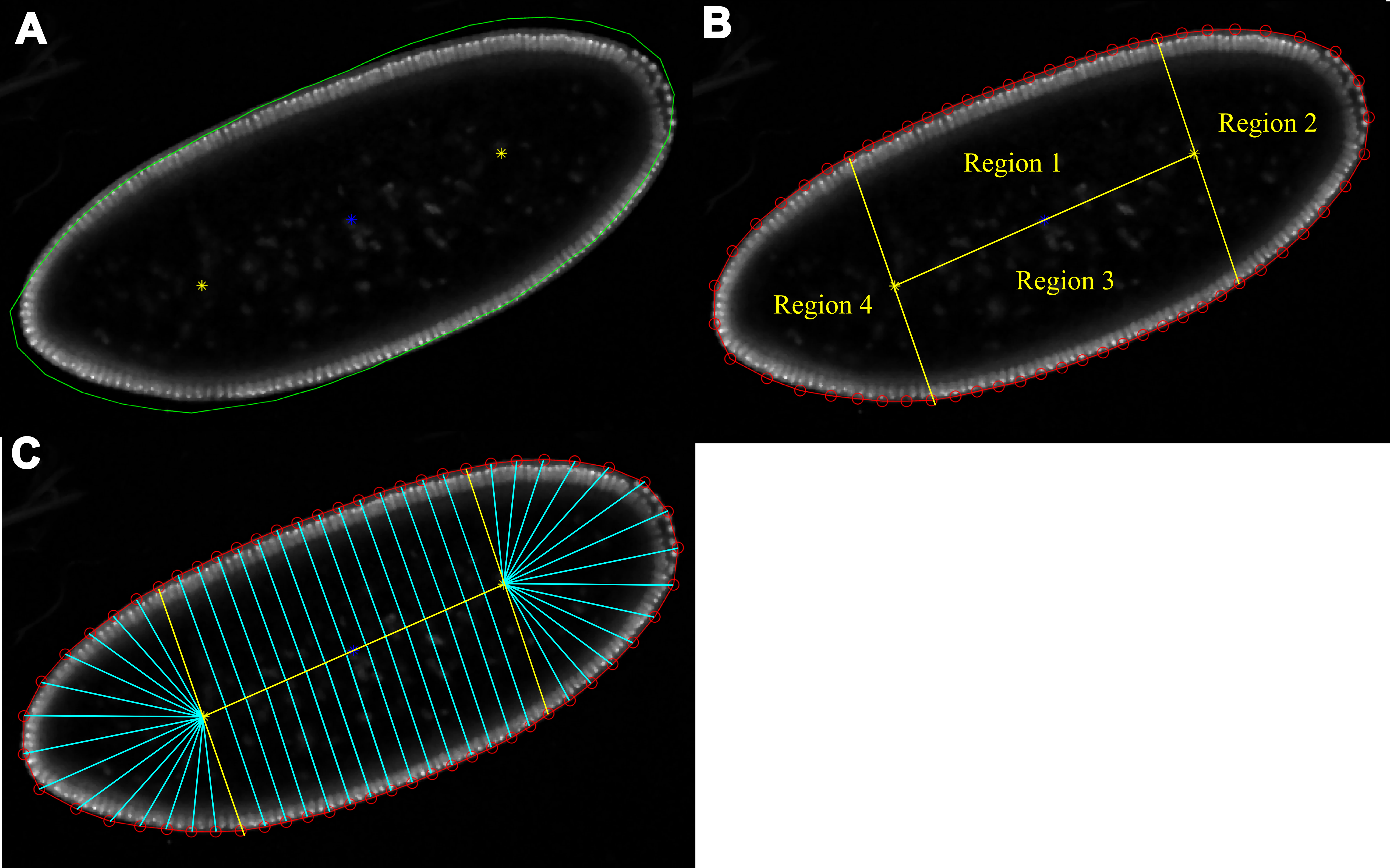


**Fig. S5:** Finding nuclei in embryo image. (A) The embryo (whose center point [blue star] has been found using previous methods) is then fit to an ellipse (green outline) with two foci (yellow stars). (B) Using the foci the image is divided into four regions. (C) Each of these regions is divided into 15 regions where the outside edge of each segment is determined (red curve). In the first two regions (regions 1 and 3 in Fig. S5), the boundary of the embryo was found by dividing the regions into 15 smaller rectangles (rather than slices as described above). This corresponded to the trunk region of the embryo. In the second two regions (regions 2 and 4 in Fig. S5), the boundary was found by dividing the regions up into 15 slices centered at the ellipse focus.

Once this updated boundary was found, an inner boundary was constructed by moving the 60 updated boundary points inward by 30 pixels in the direction of the local normal. This defined 60 quadrilaterals that encompass the outer periphery of the embryo. These quadrilaterals were laid on top of the image slice corresponding to the mid sagittal plane of the embryo, and each of the three color channels were unrolled using a affine transformation on the 60 quadrilaterals to result in 60 rectangles (see Liberman et al., 2009; Trisnadi et al., 2013 for more information).

**References**

References

1. Papatsenko D, Levine M: **The *Drosophila* gap gene network is composed of two parallel toggle switches.** PLoS One 2011, **6**(7):e21145.

2. Runarsson TP, Yao X: **Search biases in constrained evolutionary optimization.** IEEE Transactions on System, Man, and Cybernetics - Part C 2005, **35**(2):233-243.

3. Runarsson T, Yao X: **Stochastic ranking for constrained evolutionary optimization.** IEEE Trans Evol Comput 2000, **4**(3):284-294.

4. Liberman LM, Reeves GT, Stathopoulos A: **Quantitative imaging of the Dorsal nuclear gradient reveals limitations to threshold-dependent patterning in *Drosophila*.** Proc Natl Acad Sci U S A 2009, **106**(52):22317-22322.

5. Trisnadi N, Altinok A, Stathopoulos, A and Reeves,GT.: **Image analysis and empirical modeling of gene and protein expression.** Methods 2013, **62**(1):68-78.
